# Impact of obstructive sleep apnea on arrhythmic risk in ischemic heart disease and hypertrophic cardiomyopathy: insights from a large national cohort

**DOI:** 10.3389/fcvm.2026.1793921

**Published:** 2026-07-29

**Authors:** Qingqing Li, Meijin Zhang, Jingjing Gong

**Affiliations:** 1 Department of Cardiology, The First Affiliated Hospital of Fujian Medical University, Fuzhou, China; 2 Department of Cardiology, National Regional Medical Center, Binhai Campus of the First Affiliated Hospital of Fujian Medical University, Fuzhou, China; 3 Clinical Research Center for Metabolic Heart Disease of Fujian Province, Fuzhou, China; 4 Key Laboratory of Metabolic Heart Disease in Fujian Province, Fuzhou, China

**Keywords:** atrial fibrillation, cardiac arrest, hypertrophic cardiomyopathy, ischemic heart disease, obstructive sleep apnea, ventricular fibrillation

## Abstract

**Background:**

Obstructive sleep apnea (OSA) is a prevalent sleep-disordered breathing condition associated with atrial fibrillation (AF) and cardiovascular morbidity. Its impact on life-threatening arrhythmic events, including cardiac arrest (CA) and ventricular fibrillation (VF), in patients with ischemic heart disease (IHD) and hypertrophic cardiomyopathy (HCM) remains incompletely understood.

**Objectives:**

We aimed to evaluate the association between OSA and arrhythmic outcomes in patients with IHD or HCM and to explore the potential role of AF as an intermediate pathway.

**Methods:**

Using the National Inpatient Sample (2016–2020), we identified adults with IHD or HCM. OSA and arrhythmic events were defined by ICD-10 codes. Baseline characteristics were compared, and 1:1 propensity score matching (PSM) was performed. Multivariate logistic regression assessed the relationship between OSA and AF, AF and the composite endpoint of CA/VF, and the combined effect of OSA and AF in hierarchical models. Odds ratios (ORs) with 95% confidence intervals (CIs) were reported.

**Results:**

Among 20,590 patients, 3,974 (19.3%) had OSA. OSA was independently associated with AF (OR 1.37, 95% CI 1.15–1.62, *p* < 0.001). AF was a strong predictor of CA/VF (OR 13.88, 95% CI 10.70–18.01, *p* < 0.001). In hierarchical models, the effect of OSA on CA/VF was attenuated and non-significant (OR 1.14, 95% CI 0.96–1.36, *p* = 0.13), whereas AF remained robustly associated.

**Conclusions:**

In patients with IHD or HCM, OSA was associated with a higher prevalence of AF, which may represent an important intermediate pathway associated with life-threatening arrhythmic events.

## 1 Introduction

Obstructive sleep apnea (OSA) is a common sleep-related breathing disorder characterized by recurrent upper airway collapse during sleep, leading to intermittent hypoxia, sympathetic activation, and sleep fragmentation (1). OSA has been increasingly recognized as a major contributor to cardiovascular morbidity, including hypertension, coronary artery disease (CAD), atrial fibrillation (AF), and sudden cardiac death (2). Mechanistically, intermittent hypoxia and intrathoracic pressure swings promote myocardial stress, electrical remodeling, and arrhythmogenesis, particularly in patients with preexisting structural heart disease (3).

Patients with ischemic heart disease and hypertrophic cardiomyopathy (HCM) represent populations at particularly high risk for arrhythmic events (4). In CAD, OSA may exacerbate myocardial ischemia and increase susceptibility to ventricular arrhythmias. In HCM, characterized by left ventricular hypertrophy and fibrosis, the arrhythmogenic substrate may be further amplified by OSA-induced nocturnal hypoxemia and hemodynamic stress (5). Despite growing recognition of these interactions, evidence quantifying the arrhythmic impact of OSA in these high-risk populations remains limited, particularly in large, nationally representative cohorts.

Moreover, while OSA has been linked to atrial arrhythmias such as AF, its relationship with life-threatening ventricular arrhythmias, including cardiac arrest and ventricular fibrillation, is less well characterized (6). Understanding whether OSA independently increases arrhythmic risk, and whether AF may represent an intermediate pathway in this relationship, is critical for risk stratification and targeted interventions in these vulnerable patients (7).

In this study, we evaluated the impact of obstructive sleep apnea (OSA) on arrhythmic outcomes in patients with ischemic heart disease and hypertrophic cardiomyopathy using a large, nationally representative cohort.

## 2 Materials and methods

### 2.1 Study design and data source

This retrospective cohort study utilized data from the National Inpatient Sample (NIS) from 2016 to 2020, a publicly available, all-payer inpatient healthcare database in the United States (8). The NIS provides detailed patient-level information, including demographics, diagnoses, comorbidities, procedures, and discharge outcomes. Diagnoses and procedures were identified using ICD-10-CM and ICD-10-PCS codes. The use of de-identified, publicly available data rendered this study exempt from institutional review board approval.

### 2.2 Study population

We included adult patients (≥18 years) with a primary or secondary diagnosis of ischemic heart disease (IHD) or hypertrophic cardiomyopathy (HCM). The presence of obstructive sleep apnea (OSA) was identified using ICD-10 codes (Supplementary Table 1). Patients were excluded if key demographic, comorbidity, or outcome data were missing.

### 2.3 Exposures and covariates

The primary exposure of interest was OSA. Baseline patient characteristics included age, sex, race/ethnicity, and comorbidities such as hypertension, diabetes mellitus, hyperlipidemia, obesity, obstructive lung disease, renal and liver disease, and smoking or alcohol use. Clinical covariates also included systolic heart failure (defined as left ventricular ejection fraction <50%), syncope, prior myocardial infarction, atrial fibrillation (AF), AF ablation, use of implantable cardioverter-defibrillators (ICDs), and family history of sudden cardiac death (SCD).

### 2.4 Outcomes

The primary outcome was a composite endpoint defined as the presence of either cardiac arrest (CA) or ventricular fibrillation (VF) (CA/VF) during hospitalization, identified using ICD-10 diagnostic codes. We selected this composite outcome *a priori* to capture severe life-threatening arrhythmic events and to improve statistical power given the relatively low frequency of individual events. Cardiac arrest and ventricular fibrillation were not required to occur simultaneously, and patients meeting criteria for either diagnosis were included in the composite endpoint. Secondary analyses explored the potential role of AF as an intermediate pathway associated with the relationship between OSA and the composite arrhythmic outcome.

### 2.5 Statistical analysis

Continuous variables were summarized as mean ± standard deviation (SD) and compared using t-tests, whereas categorical variables were expressed as counts and percentages and compared using chi-square tests. To account for potential confounding and baseline imbalances between patients with and without OSA, exact 1:1 propensity score matching (PSM) was performed based on demographic characteristics, comorbidities, prior myocardial infarction, AF, systolic heart failure, family history of SCD, and use of ICDs. Adequacy of matching was assessed using standardized mean differences, with values <0.1 indicating acceptable balance and was visualized using love plots (Supplementary Figure 1).

Univariate and multivariate logistic regression analyses were conducted to evaluate the effects of OSA on AF, AF on the composite CA/VF endpoint, and the combined effect of OSA and AF on CA/VF in hierarchical models adjusting for relevant covariates. Odds ratios (ORs) with 95% confidence intervals (CIs) were reported for both the full and propensity-matched cohorts. Sensitivity analyses included comparisons before and after matching, and AF was evaluated as a potential intermediate pathway in the association between OSA and CA/VF.

All statistical analyses were performed using R (version 4.4.2) with appropriate packages for propensity score matching and regression modeling. A two-tailed *p*-value <0.05 was considered statistically significant.

## 3 Results

### 3.1 Baseline characteristics

A total of 20,590 patients with ischemic heart disease or hypertrophic cardiomyopathy were included, of whom 3,974 (19.3%) had a diagnosis of obstructive sleep apnea (OSA). Patients with OSA were younger (mean age 66.44 ± 11.75 vs. 70.07 ± 13.28 years, *p* < 0.001) and less frequently female (42.98% vs. 56.64%, *p* < 0.001) compared to those without OSA. Comorbidities including hypertension, diabetes, hyperlipidemia, obesity, obstructive lung disease, and renal disease were more prevalent among patients with OSA (all *p* < 0.001). Conversely, syncope was less frequent in the OSA cohort (1.81% vs. 3.10%, *p* < 0.001). Rates of prior myocardial infarction, liver disease, and family history of sudden cardiac death did not differ significantly between groups; alcohol use was less frequent in the OSA group (2.59% vs. 3.40%, *p* = 0.01) (Table 1).

**Table 1:** Baseline characteristics.

Characteristics	Crude			
	Total (*n* = 20,590)	OSA (*n* = 3,974)	No OSA (*n* = 16,616)	*p*-value
Age – Mean (SD)	69.37 ± 13.08	66.44 ± 11.75	70.07 ± 13.28	<0.001
Female, *n* (%)	11,120 (54.01%)	1,708 (42.98%)	9,412 (56.64%)	<0.001
Race^a^, *n* (%) White Black Hispanic Asian or Pacific Islander Native American Other	13,924 (69.47%) 3,904 (19.48%) 1,112 (5.55%) 564 (2.81%) 64 (0.32%) 474 (2.37%)	2,830 (73.41%) 701 (18.18%) 190 (4.93%) 57 (1.48%) 19 (0.49%) 58 (1.50%)	11,094 (68.54%) 3,203 (19.79%) 922 (5.70%) 507 (3.13%) 45 (0.28%) 416 (2.57%)	<0.001
Hypertension, *n* (%)	18,257 (88.67%)	3,646 (91.75%)	14,611 (87.93%)	<0.001
Diabetes, *n* (%)	7,514 (36.49%)	1,873 (47.13%)	5,641 (33.95%)	<0.001
Hyperlipidemia, *n* (%)	13,705 (66.56%)	2,827 (71.14%)	10,878 (65.47%)	<0.001
Obstructive lung disease, *n* (%)	6,980 (33.90%)	1,749 (44.01%)	5,231 (31.48%)	<0.001
Peripheral vascular disease, *n* (%)	2,596 (12.61%)	437 (11.00%)	2,159 (12.99%)	0.001
Active Smoking, *n* (%)	9,056 (43.98%)	1,788 (44.99%)	7,268 (43.74%)	0.15
Renal disease, *n* (%)	6,938 (33.70%)	1,515 (38.12%)	5,423 (32.64%)	<0.001
Liver disease, *n* (%)	1,261 (6.12%)	254 (6.39%)	1,007 (6.06%)	0.43
Alcohol use, *n* (%)	668 (3.24%)	103 (2.59%)	565 (3.4%)	0.01
Hyperthyroidism, *n* (%)	179 (0.87%)	36 (0.91%)	143 (0.86%)	0.78
Obesity, *n* (%)	4,363 (21.19%)	1,729 (43.51%)	2,634 (15.85%)	<0.001
Atrial Fibrillation, *n* (%)	9,796 (47.58%)	2,129 (53.57%)	7,667 (46.14%)	<0.001
AF ablation, *n* (%)	265 (1.29%)	70 (1.76%)	195 (1.17%)	0.003
Previous MI, *n* (%)	5,336 (25.92%)	1,066 (26.82%)	4,270 (25.70%)	0.16
Family history of sudden cardiac death, *n* (%)	85 (0.41%)	21 (0.53%)	64 (0.39%)	0.21
Syncope, *n* (%)	587 (2.85%)	72 (1.81%)	515 (3.1%)	<0.001
Use of ICDs, *n* (%)	712 (3.46%)	151 (3.80%)	561 (3.38%)	0.19
Systolic Heart Failure, *n* (%)	2,815 (13.67%)	623 (15.68%)	2,192 (13.19%)	<0.001

ICDs, implantable cardioverter-defibrillators; OSA, obstructive sleep apnea; MI, myocardial infarction; AF, atrial fibrillation.

There were 548 (2.66%) patients with missing race data.

### 3.2 Clinical outcomes before and after propensity matching

The composite endpoint of cardiac arrest (CA) or ventricular fibrillation (VF) occurred more frequently in patients with OSA before propensity matching (4.71% vs. 3.30%, OR 1.44, 95% CI 1.22–1.71, *p* < 0.001). After 1:1 propensity score matching, the difference was attenuated and did not reach statistical significance (4.72% vs. 3.83%, OR 1.24, 95% CI 0.99–1.55, *p* = 0.05) (Table 2).

**Table 2:** Clinical outcomes between patients with and without OSA.

Outcomes		OSA	No OSA	OR (95% CI)	*p*-value
Cardiac arrest and ventricular fibrillation, *n* (%)	Before PSM	(*n* = 3,974) 187 (4.71%)	(*n* = 16,616) 549 (3.30%)	1.44 (1.22 to 1.71)	<0.001
	After PSM	(*n* = 3,860) 182 (4.72%)	(*n* = 3,860) 148 (3.83%)	1.24 (0.99 to 1.55)	0.05

OSA, obstructive sleep apnea; PSM, propensity score matching; OR, odds ratio.

### 3.3 Multivariate logistic regression analyses

In multivariate logistic regression models adjusting for age, sex, syncope, systolic heart failure, use of implantable cardioverter-defibrillators (ICDs), and family history of sudden cardiac death, obstructive sleep apnea (OSA) was independently associated with an increased odds of atrial fibrillation (AF) (OR 1.37, 95% CI 1.15–1.62, *p* < 0.001) (Table 3). Similarly, AF was strongly associated with the composite arrhythmic endpoint of cardiac arrest or ventricular fibrillation (CA/VF) (OR 13.88, 95% CI 10.70–18.01, *p* < 0.001) (Table 4).

**Table 3:** Multivariate logistic regression analysis of factors associated with atrial fibrillation.

Variable	Univariate analysis OR (*p*-value)	Multivariate analysis OR (*p*-value)
Age	0.99 [0.988–0.999] (*p* = 0.03)	0.993 [0.987–0.999] (*p* = 0.03)
Female sex	0.81 [0.70–0.95] (*p* = 0.008)	0.81 [0.69–0.94] (*p* = 0.006)
Obstructive sleep apnea	1.36 [1.14–1.61] (*p* = 0.001)	1.37 [1.15–1.62] (*p* < 0.001)
Family history of sudden cardiac death	1.18 [0.47–2.97] (*p* = 0.72)	–
Syncope	0.61 [0.35–1.04] (*p* = 0.07)	–
Systolic heart failure	1.18 [0.96–1.44] (*p* = 0.11)	–
Use of ICDs	2.24 [1.68–2.99] (*p* < 0.001)	2.23 [1.68–2.97] (*p* < 0.001)

ICD, implantable cardio defibrillator; LVEF, left ventricular ejection fraction; OSA, obstructive sleep apnea; AF, atrial fibrillation.

### 3.4 Hierarchical analysis: exploring the potential role of AF as an intermediate pathway

To explore the potential role of AF as an intermediate pathway in the association between OSA and CA/VF, hierarchical logistic regression models including both OSA and AF were constructed. In these analyses, AF remained strongly associated with CA/VF (OR 13.88, 95% CI 10.70–18.01, *p* < 0.001), whereas the effect of OSA was attenuated and no longer statistically significant (OR 1.14, 95% CI 0.96–1.36, *p* = 0.13) (Table 4).

**Table 4:** Hierarchical multivariable logistic regression models exploring AF as a potential intermediate pathway in the association between OSA and the composite endpoint (CA/VF).

Term	Odds Ratio	95% Confidence Intervals	*P* value
Model 1: The effect of OSA on AF			
OSA	1.46	1.36–1.57	<0.001
Model 2: The effect of AF on the composite endpoint (CA/VF)			
AF	14.01	10.79–18.2	<0.001
Model 3: The effect of both OSA and AF on the composite endpoint (CA/VF).			
OSA	1.14	0.96–1.36	0.13
AF	13.88	10.70–18.01	<0.001

Other covariates are not shown.

CA/VF, cardiac arrest and ventricular fibrillation; AF, atrial fibrillation; OSA, obstructive sleep apnea.

After propensity score matching, hierarchical multivariate logistic regression confirmed these findings. AF remained strongly associated with CA/VF (OR 12.09, 95% CI 8.21–17.79, *p* < 0.001), whereas OSA showed no significant association (OR 1.03, 95% CI 0.82–1.30, *p* = 0.77) (Supplementary Table 2).

## 4 Discussion

In this large national cohort study of patients with ischemic heart disease and hypertrophic cardiomyopathy (HCM), we observed that obstructive sleep apnea (OSA) was associated with a higher prevalence of atrial fibrillation (AF) and an increased crude incidence of the composite endpoint of cardiac arrest or ventricular fibrillation (CA/VF). After adjustment for demographic and clinical covariates, OSA remained significantly associated with AF but was not independently associated with CA/VF when AF was included in hierarchical models. Propensity score–matched analyses reinforced these findings: AF demonstrated a robust, independent association with arrhythmic events, whereas the association between OSA and CA/VF was attenuated. These results suggest that AF may represent an important intermediate pathway linking OSA and severe arrhythmic events. However, due to the cross-sectional nature of the NIS dataset and the absence of temporal sequencing, a formal mediating relationship cannot be established.

These findings align with a growing body of evidence characterizing OSA as a strong arrhythmogenic substrate, particularly for AF. Data from a recent meta-analysis indicate that the incidence of AF is significantly higher in patients with OSA, with estimates of up to an 88% increased risk compared with those without OSA, even after adjustment for confounders such as age and hypertension (9, 10). Pathophysiologic mechanisms linking OSA to AF include repetitive episodes of intermittent hypoxia, negative intrathoracic pressure swings, and autonomic dysregulation resulting in atrial stretch, remodeling, and electrical instability (3, 11). These mechanisms promote structural and electrical changes in the atria that facilitate AF initiation and maintenance, consistent with our observation of a strong independent association between OSA and AF in multivariate models (12, 13).

The potential role of AF as an intermediate pathway in arrhythmic risk among patients with OSA is supported by mechanistic and clinical data. In addition to serving as a substrate for strokes and heart failure, AF is a known predictor of ventricular arrhythmias and sudden cardiac events, potentially via tachycardia-induced cardiomyopathy, irregular ventricular response, and adverse neurohormonal activation (14, 15). While our study focused on CA/VF as an outcome, the robust odds ratios observed for AF in both unmatched and matched analyses suggest that AF may represent an important clinical marker or intermediate pathway associated with severe arrhythmic outcomes. However, the magnitude of this association should be interpreted cautiously. Residual confounding, coding-related misclassification, and the inability to establish temporal relationships within the NIS database may have contributed to the observed effect size. Therefore, these findings should be considered hypothesis-generating and require confirmation in prospective studies with detailed clinical and longitudinal data. This observation is clinically meaningful because it shifts emphasis from direct OSA-linked mechanisms of ventricular arrhythmogenesis toward pathways involving atrial pathology and its downstream consequences.

Our findings also reflect nuances seen in prior literature regarding OSA and sudden cardiac death (SCD). A recent systematic review and meta-analysis found inconsistent associations between OSA and SCD, with untreated OSA showing elevated risk but overall heterogeneity limiting definitive conclusions (16). This complexity underscores that OSA may not be sufficient by itself to cause serious ventricular arrhythmias in every patient; rather, its impact may be modified by underlying cardiac conditions, comorbidities, and intermediate arrhythmias such as AF (17, 18). In our cohort of patients with IHD and HCM, two populations with heightened arrhythmic substrates due to myocardial scar and hypertrophy, OSA's contribution to arrhythmic risk appears to operate through established pathways of atrial dysfunction.

From a guideline perspective, current recommendations from major cardiovascular societies recognize the high prevalence of OSA in patients with cardiac disease and the need for systematic screening, particularly in those with AF and heart failure (19, 20). For example, the American Heart Association (AHA) statement highlights that OSA is often underrecognized in patients with coronary artery disease and arrhythmias, and recommends evaluation for sleep-disordered breathing in patients with resistant hypertension, recurrent AF after cardioversion or ablation, and nocturnal arrhythmias (21). Despite this, routine integration of OSA screening and management into cardiovascular practice remains suboptimal.

Interventional studies further suggest that treating OSA may mitigate arrhythmic risk, particularly in AF populations. Recent data highlighted that continuous positive airway pressure (CPAP) therapy reduces AF recurrence after pulmonary vein isolation and cardioversion, although the magnitude of benefit can vary by patient phenotype and left atrial size (22–24). Adherence to CPAP has also been associated with attenuated cardiovascular risk in broader cohort settings (25). These data support the notion that OSA is not merely a marker of arrhythmic risk but may be a modifiable contributor, potentially altering the trajectory of AF and its downstream complications.

### 4.1 Clinical implications

Our study has important clinical implications. First, it emphasizes the need for heightened awareness of OSA in patients with ischemic heart disease and HCM, both of which predispose to complex arrhythmias. Enhanced screening for OSA in these populations, for example, using objective sleep studies rather than symptom-based questionnaires, could identify individuals at risk of AF and facilitate early intervention (26). Second, our hierarchical analysis suggests that addressing AF as a key intermediate may be critical in reducing the arrhythmic burden associated with OSA. This could include aggressive rhythm control strategies and optimized CPAP therapy in patients with coexistent OSA and AF. Third, these findings call for future prospective research and clinical trials to validate whether targeted OSA treatment, in conjunction with rhythm management, can improve hard arrhythmic endpoints such as CA/VF.

### 4.2 Limitations

Several limitations of this study merit consideration. First, the retrospective design and reliance on administrative data from the National Inpatient Sample (NIS) introduce potential misclassification and coding bias. The identification of obstructive sleep apnea (OSA), atrial fibrillation (AF), and arrhythmic events was based on ICD-10 codes, which may under- or overestimate true disease prevalence. Furthermore, OSA is frequently underdiagnosed and undercoded in routine clinical practice; therefore, patients identified through diagnostic coding may represent a subset with more clinically recognized or severe disease, potentially introducing selection bias. Second, important clinical variables that could influence arrhythmic risk, such as left atrial size, left ventricular ejection fraction beyond the binary classification of systolic heart failure, OSA severity, polysomnography-confirmed hypoxemia metrics, medication use, and adherence to therapy (CPAP or antiarrhythmics), were not available in the dataset, limiting adjustment for all potential confounders. The absence of objective measures of OSA severity, such as the apnea-hypopnea index and nocturnal oxygen desaturation burden, precluded assessment of dose-response relationships and may limit the clinical applicability of the findings. Third, although propensity score matching and multivariate analyses were applied to account for baseline differences, residual confounding cannot be excluded. In addition, the strong association observed between AF and CA/VF may partly reflect unmeasured confounders or coding-related bias that could not be fully addressed using administrative data. Consequently, the magnitude of the reported odds ratios should be interpreted with caution. Fourth, the study combined patients with ischemic heart disease (IHD) and hypertrophic cardiomyopathy (HCM) into a single analytical cohort. Although both conditions are associated with elevated arrhythmic risk and were included to evaluate the overall impact of OSA in patients with structural heart disease, they differ substantially in their underlying pathophysiology and arrhythmogenic mechanisms. The NIS database did not allow robust subgroup analyses with adequate adjustment for all relevant clinical variables. Therefore, potential differences in the association between OSA, AF, and arrhythmic outcomes across these disease populations could not be fully explored. Fifth, the primary outcome was restricted to cardiac arrest or ventricular fibrillation. Other clinically relevant arrhythmias, including ventricular tachycardia, were not evaluated and therefore the overall burden of arrhythmic events may have been underestimated. Sixth, the NIS captures inpatient hospitalizations and does not include outpatient follow-up or longitudinal arrhythmic events after discharge, which may underestimate the incidence of AF-related complications or sudden cardiac events. Finally, causality cannot be inferred from these observational data. Because the NIS does not provide temporal information regarding the onset of OSA, AF, and arrhythmic events, it is not possible to determine whether AF truly mediates the relationship between OSA and CA/VF. Therefore, the observed associations should be interpreted as hypothesis-generating rather than evidence of a causal pathway.

## 5 Conclusion

In this large national cohort of patients with ischemic heart disease and hypertrophic cardiomyopathy, OSA was associated with a significantly higher prevalence of AF, a potent predictor of arrhythmic events. Although OSA alone appeared to increase the risk of cardiac arrest or ventricular fibrillation (CA/VF) in unadjusted analyses, hierarchical and propensity score–adjusted models demonstrated that the association between OSA and CA/VF was markedly attenuated after accounting for AF. These findings suggest that AF may represent an important intermediate pathway associated with the relationship between OSA and life-threatening arrhythmias. Clinicians should recognize the arrhythmic burden associated with OSA and the importance of monitoring for AF in these high-risk populations. Future prospective studies incorporating objective OSA severity metrics, continuous rhythm monitoring, and treatment adherence are warranted to validate these observations and inform targeted interventions aimed at reducing arrhythmic risk in patients with OSA and structural heart disease.

## Data Availability

The data analyzed in this study were obtained from the Healthcare Cost and Utilization Project (HCUP) National Inpatient Sample (NIS). Owing to HCUP licensing restrictions, we are unable to share the data publicly. Requests for access should be directed to HCUP/AHRQ.
