# Supplementary material for: Impact of obstructive sleep apnea on arrhythmic risk in ischemic heart disease and hypertrophic cardiomyopathy: insights from a large national cohort
Source: Front Cardiovasc Med. 2026 Jul 29;13:1793921. doi: 10.3389/fcvm.2026.1793921 (PMC13463152; doi:10.3389/fcvm.2026.1793921)
Supplement: Supplementary file 1 [file Datasheet1.docx]

**Supplementary Figure 1:** Love plot for the nearest-matched population.

SCD: sudden cardiac death, MI: myocardial infarction. PS: propensity.

| **Variable Name** | **Codes** |
| --- | --- |
| Obstructive Hypertrophic Cardiomyopathy | I42.1 |
| Non-Obstructive Hypertrophic Cardiomyopathy | I42.2 |
| Obstructive Sleep Apnea | G47.33 |
| Personal History of Cardiac Arrest | Z86.74 |
| Family History of Sudden Cardiac Death | Z82.41 |
| Systolic heart failure | I50.1, I50.20-I50.23, I50.33, I50.40-I50.43, I50.810-I50.814, I50.82-I50.84, I50.89, I50.9; CIR019 |
| Implantable Cardioverter Defibrillator | 02H40KZ, 02H43KZ, 02H44KZ, 02H60KZ, 02H63KZ, 02H64KZ, 02H70KZ, 02H73KZ, 02H74KZ, 02HK0KZ, 02HK3KZ, 02HK4KZ, 02HL0KZ, 02HL3KZ, 02HL4KZ, 02HN0KZ, 02HN3KZ, 02HN4KZ, 0JH607Z, 0JH608Z, 0JH609Z, 0JH60FZ, 4B02XTZ, 0JH637Z, 0JH638Z, 0JH639Z |
| Cardiac arrest-Ventricular fibrillation | CIR018;146.2 |
| Atrial Fibrillation | I48.0, I48.1, I48.11, I48.19, I48.2, I48.20, I48.21, I48.91, I48.3, I48.4, I48.92 |
| Hypertension | I10.xx; I11.xx; I12.xx; I13.xx; I15.xx; I16.xx |
| Diabetes Mellitus | E08.xx–E13.xx |
| Hyperlipidemia | E78.0–E78.5 |
| Chronic Ischemic Heart Disease | I25.xx |
| Chronic Obstructive Pulmonary Disease (COPD) | J41–J47 |
| Peripheral Vascular Disease | I70.x; I71.x; I73.1; I73.8; I73.9; I77.1; I79.0; I79.2; K55.1; K55.8; K55.9; Z95.8; Z95.9 |
| Smoking Status | F17.xx; Z72.0; Z87.891 |
| Renal Disease | I12.0; I13.1; N03.2–N03.7; N05.2–N05.7; N18.x; N19.x; N25.0; Z49.0–Z49.2; Z94.0; Z99.2 |
| Chronic Liver Disease | B18.x; I85.x; I86.4; I98.2; K70.x; K71.1; K71.3–K71.5; K71.7; K72.x–K74.x; K76.0; K76.2–K76.9; Z94.4 |
| Alcohol Abuse | Z71.41; Y90.0–Y90.9; T51.8X1A–T51.9XXS; R78.0; F10.xx series |
| Hyperthyroidism | E05.00–E05.91 |
| Obesity | E66.xx (excluding E66.3) |
| Previous Myocardial Infarction | I25.2 |

**Supplementary Table 1: Variables and ICD/PCS Codes**

| Term | Odds Ratio | 95% Confidence Intervals | P value |
| --- | --- | --- | --- |
| Model 1: The effect of OSA on AF | | | |
| OSA | 1.47 | 1.34 – 1.61 | <0.001 |
| Model 2: The effect of AF on the composite endpoint (CA-VF) | | | |
| AF | 12.13 | 8.27 – 17.79 | <0.001 |
| Model 3: The effect of both OSA and AF on the composite endpoint (CA-VF). | | | |
| OSA | 1.03 | 0.82 – 1.3 | 0.77 |
| AF | 12.09 | 8.21 – 17.79 | <0.001 |

**Supplementary Table 2:** Hierarchical multivariate logistic regression models exploring AF as a mediator of the OSA–campsite endpoint (CA-VF) relationship following the PSM model analysis.

*Note: The other variables were masked.*

CA-VF: cardiac arrest and ventricular fibrillation. AF: atrial fibrillation, OSA: obstructive sleep apnea.
